# Rostrocaudal Areal Patterning of Human PSC-Derived Cortical Neurons by FGF8 Signaling

**DOI:** 10.1523/ENEURO.0368-17.2018

**Published:** 2018-04-26

**Authors:** Kent Imaizumi, Koki Fujimori, Seiji Ishii, Asako Otomo, Yasushi Hosoi, Hiroaki Miyajima, Hitoshi Warita, Masashi Aoki, Shinji Hadano, Wado Akamatsu, Hideyuki Okano

**Affiliations:** 1 Department of Physiology, Keio University School of Medicine, Shinjuku, Tokyo 160-8582, Japan; 2 Research Fellow of Japan Society for the Promotion of Science, Chiyoda, Tokyo 102-0083, Japan; 3 Department of Molecular Life Sciences, Tokai University School of Medicine, Isehara, 259-1193, Japan; 4 First Department of Medicine, Hamamatsu University School of Medicine, Hamamatsu, 431-3192, Japan; 5 Department of Neurology, Tohoku University Graduate School of Medicine, Aoba-ku, Sendai 980-8574, Japan; 6 Center for Genomic and Regenerative Medicine, Juntendo University School of Medicine, Bunkyoku, Tokyo 113-8421, Japan

**Keywords:** amyotrophic lateral sclerosis, areal patterning, fibroblast growth factor 8, pluripotent stem cell

## Abstract

The cerebral cortex is subdivided into distinct areas that have particular functions. The rostrocaudal (R-C) gradient of fibroblast growth factor 8 (FGF8) signaling defines this areal identity during neural development. In this study, we recapitulated cortical R-C patterning in human pluripotent stem cell (PSC) cultures. Modulation of FGF8 signaling appropriately regulated the R-C markers, and the patterns of global gene expression resembled those of the corresponding areas of human fetal brains. Furthermore, we demonstrated the utility of this culture system in modeling the area-specific forebrain phenotypes [presumptive upper motor neuron (UMN) phenotypes] of amyotrophic lateral sclerosis (ALS). We anticipate that our culture system will contribute to studies of human neurodevelopment and neurological disease modeling.

## Significance Statement

Although the cerebral cortex is organized into functionally unique subdivisions or areas, the areal specification has not been studied extensively in pluripotent stem cell (PSC)-based neurodevelopmental models. Here, we report a culture system to control the areal identity of PSC-derived cerebral cortical progenitors along the rostrocaudal (R-C) axis by modulating fibroblast growth factor 8 (FGF8) signaling. Treatment with FGF8 conferred rostral (the sensorimotor cortex) identity on cerebral cortical progenitors, whereas these progenitors retained caudal (the temporal lobe) identity in the absence of FGF8. By using this culture system, we succeeded in modeling area-specific forebrain phenotypes [presumptive upper motor neuron (UMN) phenotypes] of amyotrophic lateral sclerosis (ALS). This system offers a novel platform in the field of human neurodevelopment and neurologic disease modeling.

## Introduction

The cerebral cortex has a pivotal role in higher-order brain functions in humans. It is divided into discrete, specialized subdomains called areas, and its complex information-processing capability is a function of neuronal computation performed across these areas. Areal identity is initially defined during neural development and is driven by morphogens that are secreted from patterning centers (O’Leary et al., 2007). For instance, it has been demonstrated that the rostrocaudal (R-C) gradient of fibroblast growth factor 8 (FGF8) secreted from the anterior neural ridge (ANR) patterns the cortical areas (Fukuchi-Shimogori and Grove, 2001). However, the mechanism of areal patterning has mostly been studied in mouse models, and it is unclear whether the findings can be applied to human cerebral cortex development.

It is difficult to study human neurodevelopment using human neural cells directly taken from embryos because this process is ethically and technically restricted. To overcome these limitations, researchers now take advantage of human pluripotent stem cells (PSCs), including embryonic stem cells (ESCs) and induced PSCs (iPSCs). These cells have the potential to differentiate into various neural subtypes and offer *in vitro* models to study the developmental process in humans (Tao and Zhang, 2016). Indeed, human PSCs can recapitulate the regional patterning of various brain regions, including the forebrain, the midbrain, the hindbrain, and the spinal cord (Kadoshima et al., 2013; Maroof et al., 2013; Imaizumi et al., 2015; Lippmann et al., 2015; Muguruma et al., 2015; Lu et al., 2016). In this way, PSCs provide a promising tool to study human cerebral cortical area patterning. However, cortical areal identity in PSC cultures has not been extensively studied.

PSCs also have a remarkable potential to serve as *in vitro* models of neurologic diseases. Given that it is difficult to obtain patient-derived neural cells or tissues because of the limited accessibility of the brain, PSC-based recapitulation of disease phenotypes is an attractive tool for clarifying pathogenesis. When modeling neurologic diseases with PSCs, it is necessary to generate neural cells with appropriate regional identities because most neurologic diseases preferentially affect specific brain regions (Mattis and Svendsen, 2011; Marchetto and Gage, 2012; Imaizumi and Okano, 2014; Okano and Yamanaka, 2014). In terms of diseases that affect the cortex, specific areas are often selectively damaged; for example, the motor cortex is a prime target in amyotrophic lateral sclerosis (ALS). Therefore, the technology to control the areal identity of PSC-derived cortical neurons will also be helpful for *in vitro* modeling of neurologic diseases.

Here, we report a PSC-based culture system that models control of the areal identity of cerebral cortical progenitors along the R-C axis by modulating FGF8 signaling. The control of R-C identity was confirmed by analyzing the expression of the R-C markers and by comparing their transcriptome with that of human fetal brains. Furthermore, we detected area-specific forebrain phenotypes [presumptive upper motor neuron (UMN) phenotypes] of ALS by using this culture system. Our work opens up new opportunities for studies of human neurodevelopment and neurologic disease modeling.

## Materials and Methods

### Generation and culture of undifferentiated ESCs and iPSCs

Human ESCs (KhES-1, 46XX; Suemori et al., 2006), control human iPSCs (201B7, 46XX; Takahashi et al., 2007), *FUS*-mutated iPSCs (FALS-e46, 46XY; Ichiyanagi et al., 2016), and *ALS2*-mutated iPSCs (4605, 46XY) were used in this study. We generated *ALS2*-mutated iPSCs from a patient with familial ALS (ALS2; Shirakawa et al., 2009) as previously described (Seki et al., 2010). These cells were cultured on SNL murine fibroblast feeder cells in standard human ESC medium in an atmosphere containing 3% CO_2_ (Imaizumi et al., 2015).

ESCs were used in accordance with the guidelines regarding the utilization of human ESCs, with approval from the Ministry of Education, Culture, Sports, Science, and Technology of Japan and the Keio University School of Medicine Ethics Committee. All experimental procedures for iPSCs derived from patients were approved by the Keio University School of Medicine Ethics Committee (approval no. 20080016).

### Neuronal induction

Neuronal induction of ESCs/iPSCs was performed by using the neurosphere culture system as previously described (Imaizumi et al., 2015) with slight modifications. Briefly, ESCs/iPSCs were pretreated for 6 d with 3 µM SB431542 (Tocris) and 150 nM LDN193189 (StemRD). They were then dissociated and seeded at a density of 10 cells/µl in media hormone mix (MHM; Shimazaki et al., 2001; Okada et al., 2004, 2008) with selected growth factors and inhibitors under conditions of 4% O_2_/5% CO_2_. The growth factors and inhibitors included 20 ng/ml FGF-2, 1× B27 supplement without vitamin A (Invitrogen), 2 µM SB431542, 10 µM Y-27632 (Calbiochem), and 3 µM IWR-1e (Calbiochem). Defining the day on which neurosphere culture was started as day 0, cells were reseeded at 50 cells/µl in MHM with 1× B27 and 10 µM Y-27632 on day 12. The following patterning factors were also added on day 12: 50–200 ng/ml FGF8 (Peprotech) and 100 ng/ml soluble FGFR3 (Peprotech). On day 18, neurospheres were replated en bloc on coverslips coated with poly-ornithine and laminin and cultured under conditions of 5% CO_2_. The medium was changed to MHM supplemented with 1× B27.

For lower motor neuron (LMN) induction, pretreated PSCs were seeded at 10 cells/µl in MHM with 20 ng/ml FGF-2, 1× B27, 2 µM SB431542, 10 µM Y-27632, and 3 µM CHIR99021 (Stemgent), 1 µM retinoic acid (Sigma) under conditions of 4% O_2_/5% CO_2_. On day 2, 100 ng/ml Shh-C24II (R&D Systems) and 1 µM purmorphamine (Calbiochem) were added. On day 6, cells were reseeded at 10–50 cells/µl in MHM with 20 ng/ml FGF-2, 1× B27, 10 µM Y-27632, 1 µM retinoic acid, and 1 µM purmorphamine. On day 12, neurospheres were replated and cultured under conditions of 5% CO_2_. The medium was changed to MHM supplemented with 1× B27 and 1 µM DAPT (Sigma).

### Quantitative RT-PCR

Total RNA was isolated with the RNeasy Mini kit (QIAGEN) with DNase I treatment, and cDNA was prepared by using a ReverTraAce qPCR RT kit (Toyobo). The qRT-PCR analysis was performed with SYBR premix Ex Taq II (Takara Bio) on a ViiA 7 Real-Time PCR System (Applied Biosystems). Values were normalized to *ACTB*. Reactions were conducted in duplicate, and data were analyzed by using the comparative (ΔΔCt) method. Relative expression levels are presented as geometric means ± geometric SEM. The primers used for qPCR were as follows: *ACTB*, forward 5’-TGAAGTGTGACGTGGACATC-3’, reverse 5’-GGAGGAGCAATGATCTTGAT-3’; *SP8*, forward 5’-TTCTAGGGCGTGGTGCTTG-3’, reverse 5’-GAAGAGGACGAGGAGCGTTT-3’; *PEA3*, forward 5’-CTCGCTCCGATACTATTATG-3’, reverse 5’-CTCATCCAAGTGGGACAAAG-3’; *COUP-TFI*, forward 5’-AAGCCATCGTGCTGTTCAC-3’, reverse 5’-GCTCCTCACGTACTCCTCCA-3’; and *FGFR3*, forward 5’-GCCTCCTCGGAGTCCTTG-3’, reverse 5’-CGAAGACCAACTGCTCCTG-3’.

### Immunocytochemistry

Cells were fixed with 4% paraformaldehyde for 15 min at room temperature and then washed three times with PBS. After incubating with blocking buffer (PBS containing 5% normal fetal bovine serum and 0.3% Triton X-100) for 1 h at room temperature, the cells were incubated for 1–2 d at 4°C with primary antibodies at the following dilutions: cleaved CASPASE3 (rabbit, CST, 9661, 1:500), COUP-TFI (mouse, Perseus, PP-H8132-00, 1:1000), CTIP2 (rat, Abcam, ab18465, 1:200), HB9 (mouse IgG1, DSHB, 81.5C10, 1:250), FOXP2 (goat, Santa Cruz Biotechnology, sc-21069, 1:500), OTX1 (mouse, DSHB, 5F5, 1:5000), SOX1 (goat, R&D Systems, AF3369, 1:500), SP8 (goat, Santa Cruz Biotechnology, sc-104661, 1:500), and βIII-tubulin (mouse IgG2b, Sigma, T8660, 1:500). The cells were again washed three times with PBS and incubated with secondary antibodies conjugated with Alexa Fluor 488, Alexa Fluor 555, or Alexa Fluor 647 (Life Technologies) and Hoechst33342 (Dojindo Laboratories) for 1 h at room temperature. After washing three times with PBS and once with distilled water, samples were mounted on slides and examined by using a LSM-710 confocal laser-scanning microscope (Carl Zeiss). Images of single confocal planes are presented.

### Single-cell intensity analysis

Neurospheres on day 18 were dissociated into single cells and re-plated on coverslips for 5 h; then, they were fixed, immunolabeled, and imaged as described above. Randomly selected three representative images were analyzed by ImageJ. First, the nuclear areas were identified by Hoechst staining that was larger than 50 µm^2^ in surface area and with intensity levels that were typical and lower than the threshold brightness of pyknotic cells. Next, the eight-bit grayscale intensity values of the intended markers were measured in each nuclear area.

### Cleaved CASPASE3 analysis

Stained coverslips were imaged on the high-content cellular analysis system IN Cell Analyzer 6000 (GE Health care). Analysis using IN Cell Developer Toolbox v1.9 (GE Health care) began by identifying intact nuclei stained by Hoechst dye, which were defined as traced nuclei that were larger than 50 µm^2^ in surface area and with intensity levels that were typical and lower than the threshold brightness of pyknotic cells. Each traced nuclear region was then expanded by 50% to mark the cell soma region and cross-referenced with neuronal subtype markers (HB9, CTIP2, OTX1, FOXP2, and SOX1). Using the traced images for each cell, the number of the cleaved CASPASE3-positive products within the specific marker-positive or -negative cells was quantified; then, its ratio to the number of the specific marker-positive or marker-negative cells was reported.

### Microarray analysis

Total RNA from neurospheres on day 18 was extracted by RNeasy Micro kit (QIAGEN). A total of 20 ng of total RNA was converted into amplified cDNA by using Ovation Pico WTA System V2 (NuGEN) and labeled by using the SureTag Complete DNA Labeling kit (Agilent). The labeled cDNA was hybridized to SurePrint G3 Human GE v3 8 × 60K Microarrays (Agilent). The scanned images were analyzed with Feature Extraction Software 12.0.3.1 (Agilent) using default parameters to obtain background-subtracted and spatially detrended processed signal intensities. Expression was quantile normalized, and corrected for chip batch effect via ComBat (Johnson et al., 2007). Data were log-transformed, and ANOVA with error variance averaging was performed with the NIA Array Analysis Tool (Sharov et al., 2005). We used the maximum of actual error variance and error variance averaged across 500 genes with similar average expression as the denominator of F-statistic. Probes with top 1% of error variances were not used for the error variance averaging. *F* statistic is then used to estimate the *p* value according to theoretical *F* distribution.

The microarray dataset has been deposited in the NCBI Gene Expression Omnibus and is accessible through GEO series accession number GSE111106.

### Correlation analysis between different gene expression datasets

The microarray data for 9–11 post-conception weeks (pcw) macro-dissected human fetal brains (Ip et al., 2010) were downloaded from ArrayExpress (E-MEXP-2700), normalized using fRMA (McCall et al., 2010). The RNA-seq data (FPM values) of 12 and 13 pcw micro-dissected human fetal brains were obtained from the BrainSpan database (BrainSpan, 2017) and quantile normalized. We compared our microarray dataset with these datasets with ExAtlas software (Sharov et al., 2015). First, data were preprocessed by log-transformation and removing outliers (*z* value ≥ 8). We performed ANOVA in each dataset with error variance averaging as described above. In the case of our microarray dataset, all samples were analyzed as individual factors, and the error variance is estimated based on the half-normal probability plot method. The *p* values were transformed to false discovery rate (FDR) using the Benjamini–Hochberg method. We identified genes with significant change of expression (FDR ≤ 0.05, fold change ≥ 4) in each dataset, estimated gene expression change relative to median expression, and calculated a z value of Pearson's correlation for the subset of common significant genes. Clustering analysis was performed using complete linkage clustering and Euclidean distance.

### Experimental design and statistical analysis

All data were expressed as the mean ± SEM. Statistical analyses were performed using one-way ANOVA followed by *post hoc* Dunnett’s or Tukey’s test for multiple comparisons. ANOVA with error variance averaging was performed in the transcriptome analysis as described above.

## Results

### Expression change of R-C marker genes by modulating FGF8 signaling

**Figure 1.**
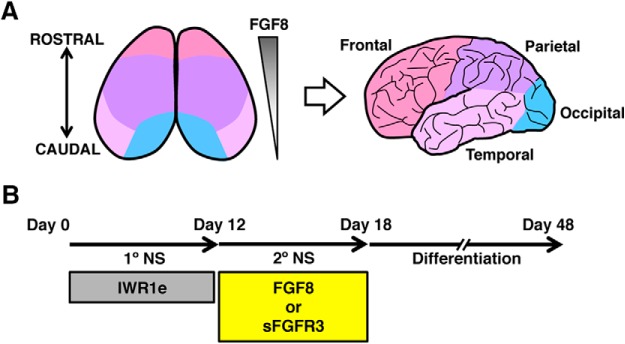
Strategy for controlling the areal identity of PSC-derived neurons. ***A***, Schematic diagram of areal patterning in the cerebral cortex. The R-C gradient of FGF8 signaling determines areal identity. ***B***, Overview of the culture protocol. FGF8 modulators (FGF8 and sFGFR3) were added at the secondary neurosphere stage.

The gradient of FGF8 signaling along the R-C axis establishes the areal identity in the developing cerebral cortex in mice (Fukuchi-Shimogori and Grove, 2001). We hypothesized that the R-C identity of neural progenitors differentiated from PSCs can be controlled by regulating FGF8 signaling, leading to the establishment of areal identity across the frontal, parietal, temporal, and occipital lobes (Fig. 1*A*).

We previously demonstrated that PSCs acquire cerebral cortical identity by inhibiting Wnt signaling during neural induction (Imaizumi et al., 2015). We used this protocol and generated neurospheres with the cerebral cortical identity derived from ESCs by treatment with the Wnt inhibitor IWR1e. FGF8 signaling was then modulated by applying recombinant FGF8 protein or soluble FGF receptor 3 (sFGFR3), which sequesters endogenous FGF8 (Fig. 1*B*).

**Figure 2.**
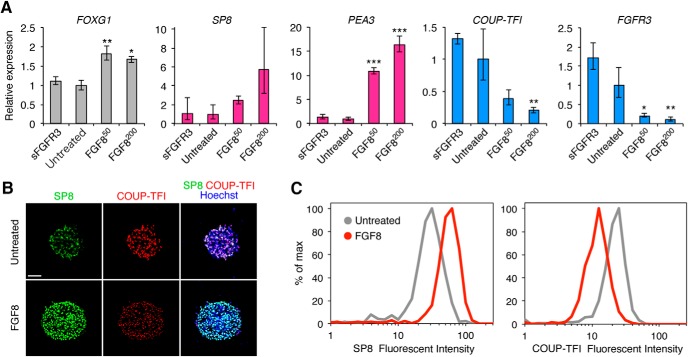
Effect of FGF8 on R-C marker expression. ***A***, qRT-PCR analysis of ESC-derived neurospheres for R-C marker expression (*n* = 3; mean ± SEM; ***p < 0.001; **p < 0.01; *p < 0.05; ANOVA with Dunnett’s test). The concentration (ng/ml) of FGF8 is presented as a superscript. ***B***, Representative immunofluorescent images for R-C markers (scale bar = 50 µm). ***C***, Histogram showing the distribution of the immunofluorescent intensity of SP8 and COUP-TFI measured at the single-cell level.

The treatment with FGF8 or sFGFR3 maintained *FOXG1* expression, indicating that fluctuations in FGF8 signaling levels did not alter the cortical identity; rather, FGF8 slightly upregulated *FOXG1* expression, as has previously been shown in mouse embryos (Shimamura and Rubenstein, 1997; Fig. 2*A*). As FGF8 signaling was activated, the rostral markers *SP8* and *PEA3* (Fukuchi-Shimogori and Grove, 2003; Sahara et al., 2007) were highly expressed, whereas the caudal markers *COUP-TFI* and *FGFR3* (Zhou et al., 2001; Hébert et al., 2003) were downregulated (Fig. 2*A*). These gene expression changes were also confirmed by immunocytochemical analysis for the SP8 and COUP-TFI proteins (Fig. 2*B*,*C*). It should be noted that untreated and FGF8-treated cells expressed low levels of SP8 and COUP-TFI, respectively. This result is consistent with the fact that the cortical regional marker genes are expressed in a graded manner, which is different from the clearly delineated, distinct expression pattern in other brain regions (Sansom and Livesey, 2009). Overall, our results suggest that R-C marker expression can be controlled by modulating FGF8 signaling during neurosphere formation from human PSCs.

### Transcriptome profiling and comparison with human fetal brains

**Figure 3.**
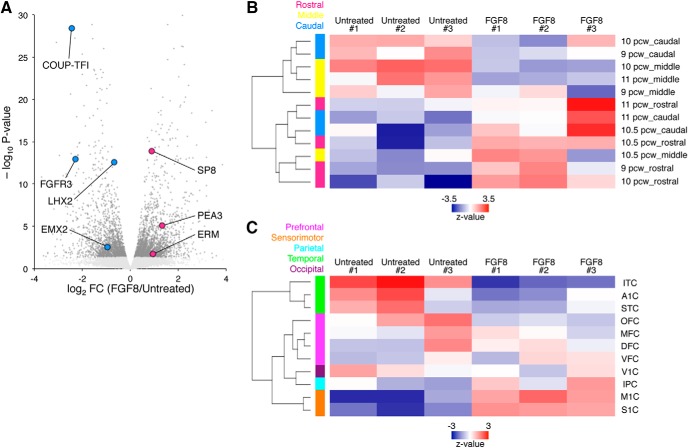
Transcriptome comparison between PSC-derived neural progenitors and human fetal brains. ***A***, Volcano plot of the expression profile of FGF8-treated cells relative to control, with differentially expressed genes (*p* < 0.05; Student’s *t* test) highlighted (dark gray). The rostral (red) and caudal (blue) markers were enriched in FGF8-treated and untreated cells, respectively. ***B***, ***C***, Correlation matrix of the global gene expression with macro-dissected (***B***) and micro-dissected (***C***) human fetal brains. FGF8-treated cells well correlated with the rostral portions of macro-dissected brains and with the sensorimotor cortex of micro-dissected brains. OFC, orbital prefrontal cortex; MFC, mediolateral prefrontal cortex; DFC, dorsolateral prefrontal cortex; VFC, ventrolateral prefrontal cortex; M1C, primary motor cortex; S1C, primary sensory cortex; IPC, inferior parietal cortex; A1C, primary auditory cortex; STC, superior temporal cortex; ITC, inferolateral temporal cortex; V1C, primary visual cortex.

To further investigate R-C identity, we performed gene expression profiling by microarray analysis. The rostral and caudal markers were enriched in FGF8-treated and untreated cells, respectively (Fig. 3*A*). A comparison of these data with those from human fetal brains dissected as 5-mm coronal slices along the R-C axis (Ip et al., 2010) showed that FGF8-treated cells more closely matched the rostral areas while showing less similarity to the middle and caudal areas (Fig. 3*B*). To characterize the areal identity in more detail, our microarray data were next compared with the RNA-seq data from micro-dissected brains of human embryos from the BrainSpan database (BrainSpan, 2017). This analysis demonstrated that untreated cells were best correlated with the temporal lobe, while FGF8-treated cells most closely resembled the sensorimotor cortex (Fig. 3*C*). Collectively, these data indicate that the global gene expression patterns of PSC-derived neural progenitors were shifted toward a rostral fate by the activation of FGF8 signaling.

### Cortical neurons with rostral identity exhibited ALS phenotypes

**Figure 4.**
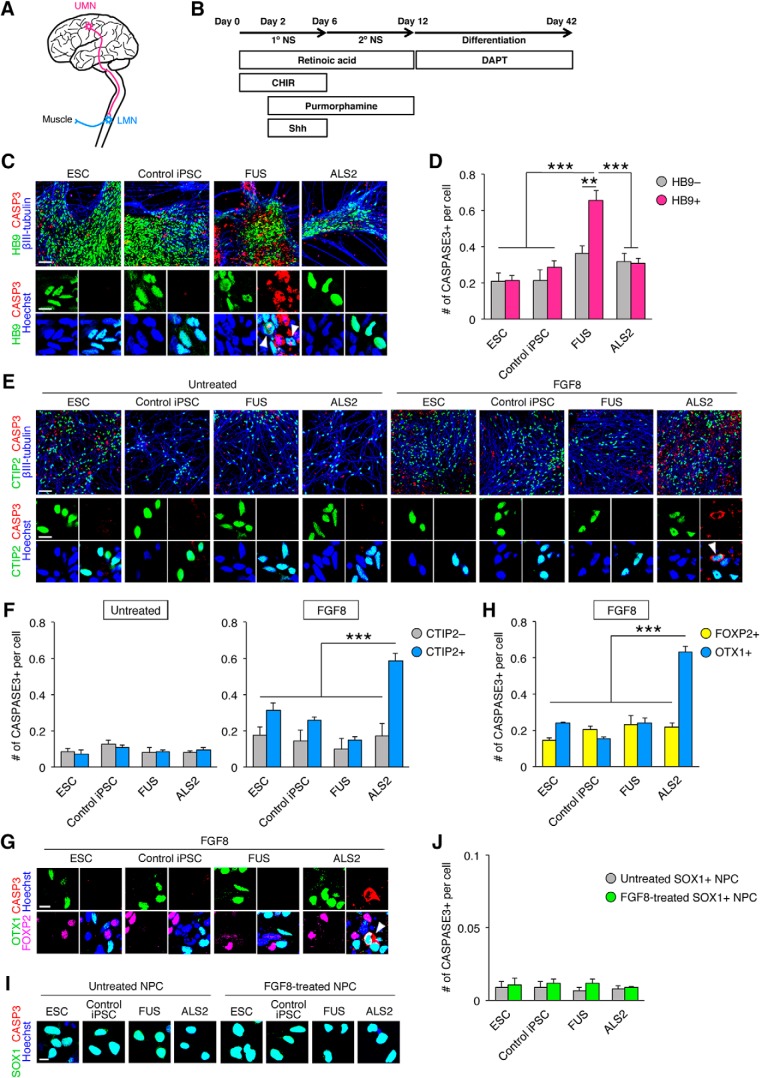
Recapitulation of ALS phenotypes in *FUS-* and *ALS2*-mutated cells. ***A***, Schematic diagram of motor neurons affected in ALS. ***B***, Overview of the LMN derivation protocol. ***C***, Representative immunofluorescent images of apoptotic LMNs at day 42 [scale bar = 50 µm (upper) and 10 µm (lower)]. Arrowheads indicate the cleaved CASPASE3-positive cells. ***D***, Quantification of the cleaved CASPASE3-positive products in LMNs at day 42 (*n* = 3; mean ± SEM; ***p < 0.001; **p < 0.01; ANOVA with Tukey’s test). HB9-positive LMNs derived from *FUS*-mutated cells selectively showed increased apoptosis. ***E***, Representative immunofluorescent images of apoptotic cortical cells at day 48 [scale bar = 50 µm (upper) and 10 µm (lower)]. Arrowheads indicate the cleaved CASPASE3-positive cells. ***F***, Quantification of the cleaved CASPASE3-positive products in untreated and FGF8-treated cortical cells at day 48 (*n* = 3–5; mean ± SEM; ***p < 0.001; ANOVA with Tukey’s test). CTIP2-positive cortical cells derived from FGF8-treated *ALS2*-mutated neurospheres selectively showed increased apoptosis. ***G***, Representative immunofluorescent images of apoptotic cortical cells at day 48 (scale bar = 10 µm). Arrowheads indicate the cleaved CASPASE3-positive cells. ***H***, Quantification of the cleaved CASPASE3-positive products in untreated and FGF8-treated cortical cells at day 48 (*n* = 4–5; mean ± SEM; ***p < 0.001; ANOVA with Tukey’s test). OTX1-positive cortical cells, but not FOXP2-positive cells, derived from FGF8-treated *ALS2*-mutated neurospheres selectively showed increased apoptosis. ***I***, Representative immunofluorescent images of SOX1/cleaved CASPASE3 in neural progenitors in neurospheres at day 18 (scale bar = 10 µm). ***J***, Quantification of the cleaved CASPASE3-positive products in untreated and FGF8-treated SOX1-positive neural progenitors in neurospheres at day 18 (*n* = 3; mean ± SEM). Selective cell death was not observed in neural progenitors.

ALS affects both UMNs and LMNs (Fig. 4*A*); however, PSC-based disease modeling has been successfully achieved only in LMN phenotypes because UMN derivation protocols have never been established (Sances et al., 2016). As our data indicated that FGF8-treated cells correspond to the primary motor cortex, we hypothesized that these cells can elucidate UMN phenotypes. To test this hypothesis, we used iPSC lines from two kinds of familial ALS: ALS2 and ALS6, carrying *ALS2* or *FUS* mutations, respectively (Shirakawa et al., 2009; Akiyama et al., 2016). These cells were differentiated into neurons by adapting the above protocol or an LMN derivation method as previously described (Imaizumi et al., 2015; Fig. 4*B*). We observed increased cleaved CASPASE3 activity in HB9-expressing LMNs derived from *FUS*-mutated iPSCs compared with those from healthy control ESC/iPSC lines, as was shown in a previous study (Ichiyanagi et al., 2016); however, such phenotypes were absent in *ALS2*-mutated cells (Fig. 4*C*,*D*). On the other hand, FGF8-treated forebrain neurons derived from *ALS2*-mutated iPSCs showed increased apoptosis, while there were no changes in untreated cells (Fig. 4*E*,*F*). *FUS*-mutated forebrain neurons did not exhibit such phenotypes in either untreated or FGF8-treated cultures. These data suggest that an *ALS2* mutation enhances apoptosis only in forebrain neurons with rostral identity. Importantly, this phenotype was observed only in cells labeled with CTIP2, a marker for Layer V subcerebral projection neurons, indicative of UMN-specific vulnerability (Molyneaux et al., 2007). The UMN selectivity of this phenotype is also supported by the fact that cells positive for OTX1, another Layer V maker, showed increased apoptosis, whereas FOXP2-positive Layer VI corticothalamic neurons did not (Molyneaux et al., 2007; Fig. 4*G*,*H*). Finally, to assess whether this cell death vulnerability is also observed in the developmental stage, SOX1-positive neural progenitors in neurospheres were analyzed for apoptosis. FGF8-treated and untreated neural progenitors displayed no changes in apoptosis (Fig. 4*I*,*J*). Overall, selective cell death was observed in Layer V cortical neurons with the rostral identity derived from *ALS2*-mutated iPSCs, suggesting that UMN phenotypes of ALS were recapitulated.

## Discussion

In this study, we established a culture system to control the R-C identity of PSC-derived cortical neurons by regulating FGF8 signaling. FGF8 activation converted the cell fate from caudal (the temporal lobe) to rostral (the sensorimotor cortex). Moreover, the area-specific forebrain phenotypes of *ALS2*-associated ALS were reproduced *in vitro* by using this system.

The gradient of morphogens, such as FGFs, Wnts, and BMPs, determines the areal identity during neural development (O’Leary et al., 2007). Of these signaling molecules, FGF8 has been most studied as a central regulator of cortical area patterning. FGF8 is secreted from ANR, which is located in the rostral-most part of the neural tube, establishes a gradient along the R-C axis, and modulates the expression of transcription factors that specify the areal identity (Fukuchi-Shimogori and Grove, 2003; Toyoda et al., 2010). Our primary aim was *in vitro* recapitulation of this mechanism by using PSCs. In fact, it has previously been reported that FGF8 treatment changes the R-C marker expression in mouse and human PSC-derived neurons (Eiraku et al., 2008; Kadoshima et al., 2013); however, these studies examined the expression of only a few markers. In contrast, we investigated the areal identity in more detail by comparing the global gene expression profile with that of fetal brains in the existing databases. Our data confirm and extend these previous reports and suggest that the areal patterning can be precisely controlled in human PSC cultures.

The protocols to produce subcerebral projection neurons in the primary motor cortex, or UMNs, from PSCs have not yet been established, hindering the prospect of modeling the pathogenesis of ALS *in vitro* (Sances et al., 2016). We challenged this problem by using our culture protocol. In these experiments, we chose *ALS2*-mutated iPSCs as a source because the mutation of *ALS2* that we used results in UMN-dominant symptoms (Shirakawa et al., 2009) and *ALS2* is also known to be a causative gene for other UMN diseases, such as primary lateral sclerosis (PLS) and hereditary spastic paraplegia (HSP; Chandran et al., 2007; Otomo et al., 2012). It is noteworthy that the observed phenotypes were both area-specific and layer specific. This double selectivity increased the reliability of our claim that UMN phenotypes can be recapitulated in our culture system. On the other hand, *FUS*-mutated cells showed only LMN phenotypes in our culture system, consistent with the fact that this patient exhibited LMN symptoms at onset, preceding that of UMN, and that these are not complicated by other forebrain-associated symptoms, such as frontotemporal dementia (FTD; Akiyama et al., 2016). These results indicate that our culture system recapitulated well the clinical manifestation of *ALS2-* and *FUS*-associated ALS. The difference in the observed phenotypes between these two types of ALS suggests that ALS pathogenesis can be divided into two groups: one predominantly affects UMNs, and the other preferentially disturbs LMNs. Although the mechanism of the selectivity of ALS phenotypes is still unclear, our system now offers new opportunities to clarify this mechanism.

Before perfect control of the areal identity of PSC-derived neurons can be demonstrated, there are some remaining issues to be resolved: (1) hodological characterization in transplantation experiments and (2) the requirement for signaling cues in addition to FGF8. The areal identity is characterized not only by gene expression patterns but also by hodological properties. In previous studies, when grafted into the mouse cortex, mouse and human PSC-derived neurons displayed specific patterns of axonal projections corresponding to the visual cortex, which indicates that these cells retain the identity of this cortical area (Gaspard et al., 2008; Espuny-Camacho et al., 2013). Such transplantation experiments will reinforce our data on areal identity.

In addition to FGF8, Wnts and BMPs also regulate areal patterning. These morphogens are secreted from the cortical hem, positioned at the medial edge of the cortex, and define areal identity (Caronia-Brown et al., 2014). A previous study showed that PSCs can be specified to differentiate into hippocampal neurons by activating Wnt and BMP signaling (Sakaguchi et al., 2015). In another study, inhibition of Wnt signaling induced rostral marker expression (Motono et al., 2016). The modulation of these signaling pathways, along with that of FGF8, will enable finer control of areal identity in PSC cultures. Additionally, other, currently unknown, signaling pathways may have an effect on areal patterning. FGF8-treated cells in our culture system acquired a rostral identity but showed similarity to the sensorimotor cortex, rather than the prefrontal cortex. Although it is probable that a higher concentration of FGF8 is necessary for the specification of the prefrontal cortex, another possibility is that additional signaling molecules are required. As the prefrontal cortex has markedly expanded through evolution, it is worth considering the hypothesis that a novel morphogen for prefrontal area patterning has emerged in human evolution. Our culture system using human PSCs will allow for the study of such human-specific neurodevelopmental processes.

## Synthesis

Reviewing Editor: Julie Andersen, Buck Institute Decisions are customarily a result of the Reviewing Editor and the peer reviewers coming together and discussing their recommendations until a consensus is reached. When revisions are invited, a fact-based synthesis statement explaining their decision and outlining what is needed to prepare a revision will be listed below. The following reviewer(s) agreed to reveal their identity: Ulrich Herget

Both the reviews and myself concur that with the revisions made, the manuscript is much improved. Although as mentioned in the Discussion similar ESC/iPSC culture systems producing a rostral neuronal population have previously been reported, the transcriptome comparison analysis in this study is a useful resource in the field.

Specific comments (combined):

Suggested changes to the text:

- Line 154: Please add that 50% expansion was done to cover the soma, as explained to the reviewers.

- Lines 197, 488: Please add the t-test that was used in Fig. 3A.

- Line 263: Please add a reference for OTX1 marking layer V.

- Lines 7, 63, 227, 288, 486: Dissected brain tissue is no longer ‘in vivo’, so I would suggest removing these words. It will remain clear that you used data from human fetal brain tissue.

Remaining general comments:

- Since ALS2 also causes other UMN diseases (PLS and HSP), as mentioned in the discussion, doesn't this approach here actually model all three diseases, instead of ALS specifically?

- Please add to the figure legends or methods if images show single confocal planes or stack projections.

Comments on figures/data:

- Fig. 3B: Please explain the many differences between the previous data and this revision in untreated #1 and #2, and FGF8 #1 and #2, which should be the same as before, as only #3 was added to each group (examples: the z-value for 10 pcw_caudal was much higher in untreated #1 compared to around 0 in #2; 10 pcw_middle was higher in untreated #1, lower in #2, and lower in FGF8 #1; 11 pcw_middle was higher in FGF8 #1; 9 pcw_middle was equally low in FGF8 #1 and #2; 11 pcw_rostral was lower in untreated #2, and higher in FGF8 #1 and 2; and 10 pcw_rostral was lower in untreated #2 in the previous data). The new macro-dissected 10.5 pcw data you added seems to actually challenge your conclusion: in the middle part, the new 10.5 pcw data is very different from 10 and 11 pcw (untreated is red at 10 and 11, but blue at 10.5; FGF8 is blue at 10 and 11, but red at 10.5). Also in the caudal part, 10 pcw (untreated red, FGF8 blue) is different from 10.5 and 11 pcw (untreated blue, FGF8 red) - please explain.

- Fig. 3C: Similar changes compared to the same brain regions in the previous data occured in 3C, where red is now white, light red turned into light blue, blue turned to red, white turned to blue, etc. Were #1 and #2 in 3B and 3C re-analyzed or were the labels mixed up?

- Fig. 4G,H: Thank you for adding this nice new data. Please add representative images as in C and E for consistency.

## Author Response

Imaizumi et al.

Point-by-point responses to the reviewers' comments

We thank the reviewers for evaluating our manuscript entitled “Rostrocaudal Areal Patterning of Human PSC-Derived Cortical Neurons by FGF8 Signaling” (eN-TNWR-0368-17R1).

We made modifications in the manuscript and the figures according to the reviewers' suggestions. Below, the reviewers' comments are presented in bold, and our responses are printed below. The changes made to the revised manuscript are highlighted in red.

-

-

Suggested changes to the text:

- Line 154: Please add that 50% expansion was done to cover the soma, as explained to the reviewers.

- Lines 197, 488: Please add the t-test that was used in Fig. 3A.

- Line 263: Please add a reference for OTX1 marking layer V.

- Lines 7, 63, 227, 288, 486: Dissected brain tissue is no longer ‘in vivo’, so I would suggest removing these words. It will remain clear that you used data from human fetal brain tissue.

> We revised the manuscript as the reviewer suggested.

Remaining general comments:

- Since ALS2 also causes other UMN diseases (PLS and HSP), as mentioned in the discussion, doesn't this approach here actually model all three diseases, instead of ALS specifically?

> ALS is distinct from the other two diseases in the way that both UMNs and LMNs are affected, whereas LMNs are not impaired in PLS and HSP. It is noted that the causative mutations in ALS2 gene are different between ALS and PLS/HSP, and the mutation used in our study cause ALS, not PLS/HSP. Thus, our data suggest that the UMN symptoms of ALS were successfully modeled.

Indeed, the ALS2 patient, from whom we derived iPSCs in this study, displayed both UMN and LMN symptoms, although UMN symptoms are dominant. However, LMN phenotypes were not recapitulated in our culture system, which indicates the possibility that the ALS2 mutation first results in the UMN damage, and then secondarily inducing the cell death of LMNs. On the other hand, FUS-mutated cells only displayed LMN phenotypes, suggesting the opposite (LMN-to-UMN) pathogenesis. To highlight these two distinct disease mechanisms, we focused on ALS in this study.

It is probable that some mechanisms in ALS are shared with PLS and HSP, and iPSCs derived from patients with these two diseases are expected to exhibit similar phenotypes in our culture system. Such experiments would be beneficial to clarify the mechanism of UMN phenotypes.

- Please add to the figure legends or methods if images show single confocal planes or stack projections.

> Images of single confocal planes are presented, and we added this information in the Materials and Methods section (Page 10, Line 8).

Comments on figures/data:

- Fig. 3B: Please explain the many differences between the previous data and this revision in untreated #1 and #2, and FGF8 #1 and #2, which should be the same as before, as only #3 was added to each group (examples: the z-value for 10 pcw_caudal was much higher in untreated #1 compared to around 0 in #2; 10 pcw_middle was higher in untreated #1, lower in #2, and lower in FGF8 #1; 11 pcw_middle was higher in FGF8 #1; 9 pcw_middle was equally low in FGF8 #1 and #2; 11 pcw_rostral was lower in untreated #2, and higher in FGF8 #1 and 2; and 10 pcw_rostral was lower in untreated #2 in the previous data). The new macro-dissected 10.5 pcw data you added seems to actually challenge your conclusion: in the middle part, the new 10.5 pcw data is very different from 10 and 11 pcw (untreated is red at 10 and 11, but blue at 10.5; FGF8 is blue at 10 and 11, but red at 10.5). Also in the caudal part, 10 pcw (untreated red, FGF8 blue) is different from 10.5 and 11 pcw (untreated blue, FGF8 red) - please explain.

- Fig. 3C: Similar changes compared to the same brain regions in the previous data occured in 3C, where red is now white, light red turned into light blue, blue turned to red, white turned to blue, etc. Were #1 and #2 in 3B and 3C re-analyzed or were the labels mixed up?

> When we combined the new data (Control#3 and FGF8#3) with the previous data, the batch effect was corrected by ComBat (Johnson et al., 2007). This approach diminishes batch-to-batch variations by the empirical Bayes model; at the same time, the expression differences may be underestimated and the statistical power is decreased. Thus, some of the differentially expressed genes in the previous dataset are now classified as non-significant in the new dataset, and the result of the transcriptome comparison analysis was changed; however, this change does not affect our conclusion that FGF8 treatment conferred rostral (sensorimotor cortex) identity, whereas caudal (temporal lobe) identity was retained in the absence of FGF8. Also, although some of macro-dissected brain samples (10.5 pcw_middle, 10.5 pcw_caudal, and 11 pcw_caudal) exhibited somewhat different trend, the tendency as a whole support our conclusion that the areal identity was shifted from caudal to rostral by FGF8.

- Fig. 4G, H: Thank you for adding this nice new data. Please add representative images as in C and E for consistency.

> The representative images are now included in Fig. 4G and 4I.

Manuscript Instructions:

- The sex of the species studied is not mentioned in the materials and methods section. Please mention ‘male’, ‘female’, ‘x males and x females’, ‘of either sex’.

> We added the description regarding the sex of donor of each ESC/iPSC line in the Materials and Methods section (Page 6, Line 2-5).
